# The Strengths and Difficulties Questionnaire Is of Clinical Significance Regarding Emotional and Behavioral Problems in 7-Year-Old Children With Familial Risk of Schizophrenia or Bipolar Disorder and Population-Based Controls the Danish High Risk and Resilience Study–VIA 7; A Population-Based Cohort Study

**DOI:** 10.3389/fpsyt.2022.861219

**Published:** 2022-05-25

**Authors:** Katrine Søborg Spang, Anne A. E. Thorup, Ditte Ellersgaard, Nicoline Hemager, Camilla Christiani, Birgitte Klee Burton, Ditte Gantriis, Aja Greve, Maja Gregersen, Ole Mors, Merete Nordentoft, Jens Richardt Møllegaard Jepsen, Carsten Obel, Kerstin J. Plessen

**Affiliations:** ^1^Mental Health Centre for Child and Adolescent Psychiatry—Research Unit, Mental Health Services Capital Region of Denmark, Copenhagen University Hospital, Copenhagen, Denmark; ^2^The Lundbeck Foundation Initiative for Integrative Psychiatric Research (iPSYCH), Aarhus, Denmark; ^3^Faculty of Health and Medical Sciences, University of Copenhagen, Copenhagen, Denmark; ^4^Copenhagen Research Centre for Mental Health—Core, Mental Health Centre Copenhagen, Copenhagen University Hospital, Mental Health Services Capital Region of Denmark, Copenhagen, Denmark; ^5^Psychosis Research Unit, Aarhus University Hospital, Aarhus, Denmark; ^6^Centre for Neuropsychiatric Schizophrenia Research & Centre for Clinical Intervention and Neuropsychiatric Schizophrenia Research, Mental Health Services—Capital Region of Denmark, Copenhagen University Hospital, Glostrup, Denmark; ^7^Department of Public Health, University of Aarhus, Aarhus, Denmark; ^8^Division of Child and Adolescent Psychiatry, Department of Psychiatry, University Hospital Lausanne, Lausanne, Switzerland

**Keywords:** SDQ, high risk, bipolar, schizophrenia, behavior, psychopathology, CBCL

## Abstract

**Background:**

Children born to parents with severe mental illness are at increased risk of mental and behavioral difficulties during childhood. We aimed to investigate the occurrence of clinically significant behavioral difficulties in 7-year-old children of parents diagnosed with schizophrenia or bipolar disorder as well as in control children by using the Strengths and Difficulties Questionnaire (SDQ). Further, we aimed to determine if the SDQ could function as a screening instrument for clinically relevant behavioral problems of children at high risk of these severe mental illnesses.

**Methods:**

By means of the Danish National Registers, we established a cohort of 522 7-year old children stratified by familial high risk for schizophrenia spectrum disorder (*N* = 202), bipolar disorder (*N* =120), and controls (*N* = 200). The child's primary caregiver completed the SDQ parent version and the Child Behavior Checklist (CBCL) while the schoolteacher completed the SDQ teacher version and the CBCL teacher equivalent; the Teachers Report Form (TRF). Finally, global functioning was assessed with the Children's Global Assessment Scale (CGAS).

**Results:**

Children with familial high risk of schizophrenia spectrum disorder or bipolar disorder have a significantly increased risk (OR = 3.8 and 2.3) of suffering clinically significant behavioral difficulties at age 7-years according to SDQ parent ratings. The SDQ discriminates with moderate to high sensitivity and high specificity between familial high-risk children with and without a psychiatric diagnosis and has overall compelling discriminatory abilities in line with the more time consuming CBCL/TRF.

Conclusions Familial high-risk children have more behavioral difficulties and more frequently at a level indicative of mental illness compared to control children as measured by the SDQ. The SDQ works well as a screening instrument for clinically relevant behavioral problems in high-risk children.

## Introduction

The single strongest risk factor for developing schizophrenia or bipolar disorder is to have a parent with the concordant illness (1). Further, the risk of developing other psychiatric disorders during childhood is also markedly increased in children of parents with severe mental illness compared with children of parents unaffected by these two disorder (2–5). Studies show that children with a first-degree relative with schizophrenia or bipolar disorder have poorer social functioning, social reciprocity, social motivation, and initiative as well as an overall higher prevalence of neurodevelopmental disorders than children without this risk factor (6–10). Thus, children with familial high risk of a severe mental illness more often require clinical intervention. Early detection of mental health problems in childhood is increasingly advocated (11). One first step toward early identification of the individual high-risk child who has or are at risk of developing mental illness, could be to identify signs of daily life behavioral difficulties indicative of mental health problems at an early age. This, preferably in an easily accessible and user-friendly way and utilizing multiple informants, especially schools, to complement and take part in the important work of early detection. By utilizing a familial high-risk study design, in which some children already have a mental illness, it is possible to assess if a dimensional assessment measure of psychopathology can identify children with a diagnosis with satisfactory accuracy, thus rendering it an applicable screening tool. If a broad band dimensional measure of psychopathology identifies the presence of mental illness with satisfactory accuracy, regular and early age assessments applying less strict cut-offs, could potentially be used to detect children before manifest disorder and thus render the possibility of intervention.

The repeatedly validated (12–16) and highly used “Strength and Difficulties Questionnaire” (SDQ) (12, 17), has the quality of being brief, free to use for non-commercial purposes and widely accepted by responders (18). The SDQ can be used to approximately determine the probability of mental illness in a population with 80 percent of a population-based sample classified as “unlikely” to have a mental illness, 10 percent as “possibly” and lastly the 10th percentile poorest scoring classified as ‘probably' having a mental illness. Utilizing these classifications the SDQ has repeatedly been found to discriminate well between clinical and non-clinical populations as well as having satisfactory predictive values in in population studies (15, 19–22). The SDQ could thus potentially serve well as a dimensional psychopathology screening tool in primary care to identify the presence of mental health problems in young children at increased risk of developing mental illness due to their familial predisposition to schizophrenia or bipolar disorder.

The primary aim of this study was to investigate if a higher proportion of children with a familial high risk of schizophrenia or bipolar disorder have behavioral difficulties above a cut-off level indicative of mental illness compared to population-based controls. We hypothesized that children with a familial high risk of schizophrenia or bipolar disorder have a significantly higher prevalence of behavioral difficulties above cut-off than children without familial high risk. We hypothesized that children with a familial high risk of bipolar disorder would have an intermediate prevalence of behavioral difficulties above cut-off compared to children of parents diagnosed with schizophrenia and control children without this familial risk factor.

Secondly, we aimed to investigate the potential of the SDQ as an assessment tool for identifying children with a mental health disorder opposed to children without any present mental health disorder in a sample of young children with familial high risk of schizophrenia or bipolar disorder and a matched control group without this familial risk factor.

To qualify an assumption of an association between an SDQ score indicative of mental illness and poorer general functioning, we sequentially assessed the difference in general functioning between children with and without potential mental illness as identified by the SDQ.

To further assess the discriminatory qualities of the SDQ, we compared them with those of the CBCL.

## Materials and Methods

### Participants

**Figure 1:**
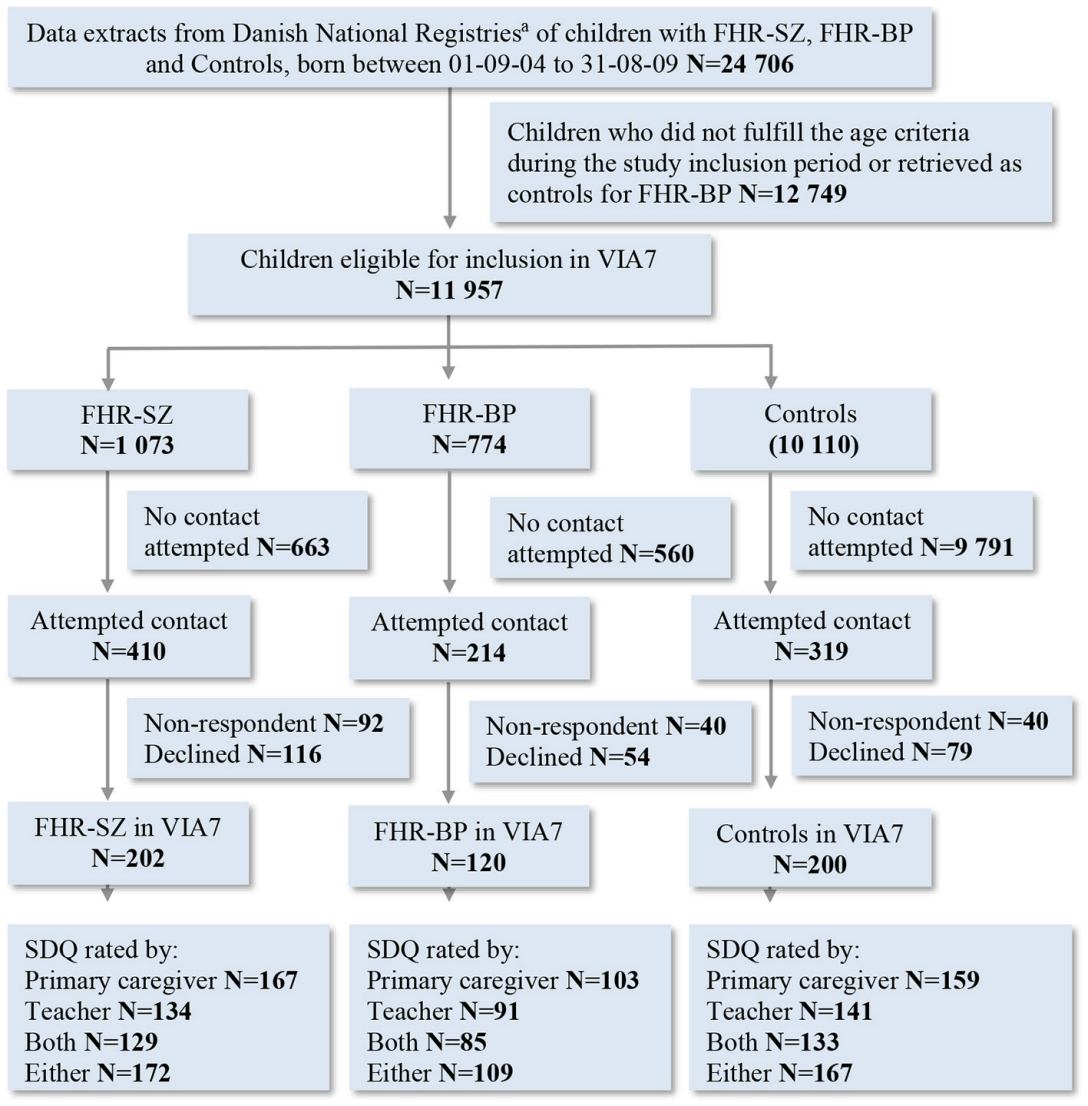
Flowchart of the inclusion of children in the Danish High Risk and Resilience Study—VIA 7 and children contributing with SDQ data. SDQ, Strengths and Difficulties questionnaire; FHR-SZ, Children of parents with schizophrenia spectrum disorders; FHR-BP, Children of parents with bipolar disorder; PBC, Population-based control children of parents with no diagnoses of schizophrenia spectrum disorders or bipolar disorder. ^a^Danish civil registration system and danish psychiatric central research register.

Four hundred forty-eight children were assessed with the SDQ; 429 children were rated by their primary caregiver; 366 children were rated by their schoolteacher and 347 were rated by both their primary caregiver and their schoolteacher (Figure 1). The primary caregiver of the child was defined as the parent or legal guardian who, at the time of the study, knew the child the best. The participating children were all part of The Danish High Risk and Resilience Study—VIA 7, hereafter referred to as the VIA 7 Study. We identified participants for the VIA 7 Study by combining information from the Danish Civil Registration System (23) and the Danish Psychiatric Central Research Register (24). The Danish National Registries allowed for identification of all 7-year-old children born and living in Denmark during the study period, while also fulfilling the VIA 7 Study inclusion criteria. The VIA 7 cohort was stratified into three groups; (1) children with familial high-risk of schizophrenia spectrum psychosis (FHR-SZ) which included children who had at least one parent diagnosed and registered with schizophrenia spectrum psychosis defined as schizophrenia, delusional disorder or schizoaffective disorder (ICD 10-codes F20, F22, and F25 or ICD 8-codes 295, 297, 298.29, 298.29, 298.89, 298.99) (25, 26); (2) children with familial high-risk of bipolar disorder (FHR-BP) which included children who had at least one parent diagnosed and registered with bipolar disorder (ICD 10-code F30 or F31 or ICD 8-codes 296.19, 296.39) (25, 26); and lastly (3) a population-based control group of children with neither parents registered with any of the diagnoses mentioned above. We matched control children to FHR-SZ children on municipality, sex, and age. We included FHR-BP children as a non-matched group. We designated children where one parent was diagnosed with bipolar disorder and the other diagnosed with schizophrenia to the FHR-SZ group as per the hierarchal principles of ICD-10. The register identified eligible children were hereafter randomly selected for the study. The VIA 7 Study was conducted between year 2013 and 2016, and the final prospective VIA 7 Study cohort consisted of 522 children: 202 FHR-SZ children, 120 FHR-BP children and 200 control children. Parents with a diagnosis of BP or SZ as specified above were classified as index-parents and the other biological parent without these diagnoses was classified as the biological non-index parent. Index parents in the FHR-SZ groups were matched with the parent of the same sex in the matched control family (27). The primary outcome measure of the current study; the SDQ, was not included from the beginning of the data collection but was introduced 6 months into the study. This resulted in a smaller total number of potential SDQ participants.

### Measures

The Strengths and Difficulties Questionnaire (SDQ) was developed as a brief behavioral screening tool to help assess child and adolescent behaviors, emotions and relationships (28). The SDQ consists of 25 questions selected on the basis of contemporary diagnostic criteria and factor analysis. The 25 items comprise five scales; four difficulties scales (Emotional Symptoms, Conduct Problems, Hyperactivity, Peer Problems) and one scale on Prosocial Behavior, and each scale consists of five items each. A Total Difficulties scale score is generated by summing the four difficulties scales. All questions are identical for the parent and teacher version and have the following response options; “*Not true,” “Somewhat true,”* or “*Certainly true”* corresponding to a score of 0, 1, and 2 or reversed scores of 2, 1, and 0 for some items. For each scale, a score between zero and ten can be obtained. For the four difficulties scales, higher scores indicate more problem behavior, whereas, for the prosocial scale, a higher score indicates better performance. The respondent is asked to consider the behavior of the child during the previous 6-month period when rating the SDQ. The SDQ is widely used internationally, and satisfactory psychometric properties as well as the clinical utility of the questionnaire, have been repeatedly confirmed (12–14, 16).

The Child Behavior Checklist school-age version (CBCL) (29) includes 118 questions on problem behavior that are rated on a Likert scale from zero (not true) to two (very true/often true). The CBCL total score is summed from the 118 problem behavior items. Further an Internalizing and an Externalizing broad-band subscale can be computed, as well as six DSM-IV oriented subscales of which we utilize the Attention-Deficit/Hyperactivity Problems scale and the Conduct Problems scale. The CBCL was completed by the primary caregiver. The Teacher's Report Form (TRF) (29) corresponds to the CBCL with minor differences and was completed by the child's schoolteacher.

Our main focus was SDQ and CBCL reports from the child's primary caregiver but we also present results of SDQ teacher reports and comparisons of discriminatory qualities of the SDQ teacher reports, against the CBCL teacher-rated equivalent, the Teachers Report Form (TRF) (29).

Best estimate lifetime DSM-IV children's psychiatric research diagnoses were ascertained through combining information from all available data on the child obtained during the test period with the semi-structured diagnostic interview; The Schedule for Affective Disorders and Schizophrenia for School-Age Children—Present and Lifetime Version (K-SADS-PL) (30). The K-SADS-PL interview was performed firstly with the primary caregiver and then with the child. For the present study, we utilized the attention-deficit/hyperactivity disorder (ADHD) diagnosis and any Axis I diagnosis of the K-SADS-PL excluding elimination disorders. We further utilized the conduct disorder and oppositional defiant disorder diagnoses to construct a disruptive behavior disorder (DBD) category. We included both definite and probable research diagnoses in the analysis.

The current level of functioning of the child was evaluated with the Children's Global Assessment Scale (CGAS) (31) as part of the K-SADS-PL interview.

Detailed results on mental health status and the severity of psychopathological dimensions are published elsewhere (3).

### Procedures

The Danish Data Protection Agency has approved the VIA 7 Study (RHP-2012-06). The study was evaluated by The Danish National Committee on Health Research Ethics. Due to the observational nature of the study, approval was not deemed necessary by the authority. We obtained informed written consent from all adult participants and custody holder(s) of the children, including a separate written consent to contact the child's schoolteacher. We asked the child's primary caregiver and the child's schoolteacher to complete the SDQ—Danish parent (SDQ-P) and teacher (SDQ-T) versions, respectively. The primary caregiver was also the main informant on all other measures concerning the child. The primary caregiver most often was a biological parent but could be a stepparent or foster parent. Trained psychologists, medical doctors and nurses performed all assessments under the supervision of a senior specialist in child neuropsychology (JRMJ) and a specialist in child and adolescent psychiatry (AAET). Child assessors were kept blinded to the risk status of the child. The complete study design of the VIA 7 Study has been described in detail elsewhere (32).

## Statistical Analyses

### Participants' Characteristics

Demographic and clinical characteristics were analyzed by one-way analysis of variance (ANOVA), chi-square test or the Mantel-Haenszel linear-by-linear test of association, as appropriate (Table 1).

**Table 1:** Characteristics of children in the Danish high risk and resilience study with an SDQ-P, their home environment, and their biological parents.

					***p*** **-value**		
	**FHR-SZ**	**FHR-BP**	**Controls**	***p*-value**	**FHR-SZ vs.** **controls**	**FHR-BP vs.** **controls**	**FHR-BP vs.** **FHR-SZ**
**Children (*****N*** **=** **429)**, ***N***	167	103	159				
% of full cohort	82.7	85.8	79.5	0.348^C^	–	–	–
Female, %	44.9	43.7	44.0	0.977^C^	–	–	–
Age at inclusion, mean (SD)	7.87 (0.20)	7.87 (0.20)	7.84 (0.19)	0.255^A^	–	–	–
CGAS^a^, mean (SD)	67.4 (15.6)	72.1 (14.8)	76.9 (13.4)	<0.0001^A^	<0.0001^A^	0.026^A^	0.033^A^
CBCL^b^ score, (*N* = 164, 102,158), *N*, mean (SD)	28.4 (20.9)	23.5 (19.8)	16.8 (14.4)	0.000^A^	0.000^A^	0.013^A^	0.101^A^
TRF^b^ score, (*N* = 141, 88,134), *N*, mean (SD)	28.5 (27.0)	22.3 (25.3)	14.7 (16.7)	0.000^A^	0.000^A^	0.054^A^	0.154^A^
Any lifetime axis I diagnosis^C^	38.3	36.9	17.6	<0.0001^C^	<0.0001^C^	<0.0001^C^	0.814^C^
Any lifetime ADHD diagnosis^d^	19.2	10.7	8.2	0.009^C^	0.004^C^	0.493^C^	0.064^C^
Any lifetime DBD diagnosis^e^	7.2	3.9	1.3	0.028^C^	0.008^C^	0.165^C^	0.264^C^
**Child's home environment**							
Living with both biological parents, %	38.3	53.4	85.5	<0.0001^C^	<0.0001^C^	<0.0001^C^	0.015^C^
Living with index^h^ parent, %	61.1	68.9	93.7	–	–	–	0.191^C^
Living with a single parent, %	35.9	31.1	9.4	<0.0001^C^	<0.0001^C^	<0.0001^C^	0.413^C^
PSP^f^ primary caregiver^g^ (*N* = 165, 103,159), mean (SD)	72.7 (14.2)	74.5 (14.0)	83.6 (9.4)	<0.0001^A^	<0.0001^A^	<0.0001^A^	0.740^A^
Primary caregiver is index	45.5	53.4	–	–	–	–	0.208
**Index**^**i**^ **Parents**, ***N***	167	102	163	–	–	–	–
Female, %	55.7	53.9	55.8	0.948^C^	–	–	–
Age at child's birth, mean (SD)	29.66 (5.85)	33.27 (7.14)	32.79 (4.66)	<0.0001^A^	<0.0001^A^	1.000^A^	<0.0001^A^
PSP^f^ (*N* = 133, 91, 156), mean (SD)	65.3 (15.7)	69.5 (13.4)	83.4 (10.3)	<0.0001^A^	<0.0001^A^	<0.0001^A^	0.055^A^
Employed/studying^h^ (*N* = 156, 96, 161), %	47.4	56.3	92.5	<0.0001^C^	<0.0001^C^	<0.0001^C^	0.174^C^
Educational information, *N*	145	96	158				
Primary/lower secondary, %	32.4	8.3	3.2	<0.0001^L^	<0.0001^L^	0.831^L^	<0.0001^L^
Upper secondary, vocational, short-cycle tertiary, %	43.4	41.7	50.0				
Bachelor degree, equivalent or higher, %	24.1	50.0	46.8				
**Non-index**^**j**^ **Parents**, ***N***	155	100	151	–	–	–	–
Female, %	43.9	46.0	43.7	0.928^C^	–	–	–
Age at child's birth, mean (SD)	30.47 (6.29)	33.11 (5.46)	33.07 (4.23)	<0.0001^A^	<0.0001^A^	1.000^A^	<0.0001^A^
PSP^f^ (*N* = 138, 86, 146), mean (SD)	75.6 (14.5)	81.5 (13.0)	85.0 (8.9)	<0.0001^A^	<0.0001^A^	0.097^A^	0.001^A^
Employed/studying^h^ (*N* = 147, 96, 148), %	74,1	88.5	95.3	<0.0001^C^	<0.0001^C^	0.0495^C^	0.006^C^
Educational information, *N*	146	93	149				
Primary/lower secondary, %	18.1	5.5	3.4	0.001^L^	0.001^L^	0.405^L^	<0.001^L^
Upper secondary, vocational, short-cycle tertiary, %	50.7	42.9	50.3				
Bachelor's degree, equivalent or higher, %	31.3	51.6	46.3				

*FHR, Familial high-risk; BP, Bipolar disorder; SZ, Schizophrenia*.

*In case information is not available for all participants an N has been added to indicate the number of individuals the result has been calculated from*.

*In the case of siblings, parent information is only included from the first included sibling in order not to count the same parent twice. This results in a lower number of parent couples than children*.

a *CGAS, children's global assessment scale (31)*.

b *CBCL, Child behavior check list; TRF, Teachers report form (29)*.

*^c^Any lifetime Axis I diagnosis*.

d *Any lifetime Axis I diagnosis*.

e *Any lifetime Axis I diagnosis*.

f *PSP, The personal and social performance scale (33)*.

g *Primary caregiver, is defined as the parent or foster parent that knows the child best and spends most time with the child*.

h *Employed or studying, is defined as being under employment (including temporary leave) or adhering to an acknowledged education for a minimum of 15 h weekly*.

i *Index parent, is defined as the parent with a diagnosis of either schizophrenia spectrum disorder or bipolar disorder and their adult matched control*.

j *Non-index parent, is defined as the biological parent without a diagnosis of schizophrenia or bipolar disorder*.

A *ANOVA test. Post-hoc one-way ANOVA with least significant difference*.

C *Chi square test*.

L *linear by linear association p-value is used when an ordinal variable has more than two categories*.

### Descriptive SDQ Mean Values and Between-Group Differences

SDQ scores were converted to Z-scores for boys and girls separately using the control group as the reference group. Hereafter Z-scores were transformed into T-scores (mean = 50 and SD = 10) with higher T-scores indicating more problem behavior on the four difficulties scales and less prosocial behavior on the prosocial scale.

The SDQ generally has highly skewed distribution of scores (34) which also was observed in the current SDQ score distributions. Consequently, we present mean raw and T-scores and the comparisons of potential group differences in T-scores were analyzed using the Welch ANOVA followed by Games-Howell *post-hoc* tests solely as descriptive illustrations of the data (Table 2).

**Table 2:** The strengths and difficulties questionnaire descriptive mean scores and standard deviations of participating children from the VIA 7 study.

**Primary caregiver** **ratings**	**FHR-SZ**		**FHR-BP**		**Controls**		**ANOVA**	**FHR-SZ vs.** **Controls**	**FHR-BP vs.** **Controls**	**FHR-SZ vs.** **FHR-BP**
	**Raw score**	**T-score**	**Raw score**	**T-score**	**Raw score**	**T-score**				
Participants, *N*	167		103		159					
	**Mean (SD)**						***p*** **-value**	***p*** **-value**		
Total difficulties	9.34 (6.49)	59.6 (16.3)	7.08 (5.66)	54.1 (13.7)	5.45 (4.32)	50.0 (10.0)	<0.001	<0.001	0.026	0.008
Emotional symptoms	2.44 (2.18)	56.8 (14.5)	2.10 (2.35)	54.5 (15.5)	1.43 (1.52)	50.0 (10.0)	<0.001	<0.001	0.025	0.454
Conduct problems	1.69 (1.65)	59.5 (17.0)	1.10 (1.40)	53.9 (14.3)	0.75 (1.15)	50.0 (10.0)	<0.001	<0.001	0.041	0.011
Hyperactivity	3.77 (2.89)	55.8 (13.3)	2.97 (2.63)	52.0 (11.9)	2.53 (2.23)	50.0 (10.0)	<0.001	<0.001	0.323	0.046
Peer problems	1.43 (1.85)	56.0 (15.9)	0.91 (1.43)	51.5 (11.9)	0.74 (1.25)	50.0 (10.0)	<0.001	<0.001	0.519	0.026
Prosocial behavior	8.28 (1.86)	52.7 (13.1)	8.73 (1.60)	49.2 (10.7)	8.67 (1.41)	50.0 (10.0)	0.039	0.093	0.810	0.045
**Teacher ratings**	**FHR-SZ**		**FHR-BP**		**Controls**		**ANOVA**	**FHR-SZ vs**. **Controls**	**FHR-BP vs**. **Controls**	**FHR-SZ vs**. **FHR-BP**
	**Raw score**	**T-score**	**Raw score**	**T-score**	**Raw score**	**T-score**				
Participants, *N*	134		91		141					
	**Mean (SD)**						***p*** **-value**	***p*** **-value**		
Total difficulties	8.31 (7.02)	57.3 (13.6)	6.75 (7.04)	54.0 (13.9)	4.67 (5.08)	50.0 (10.0)	<0.001	<0.001	0.048	0.180
Emotional symptoms	1.93 (2.07)	54.3 (11.4)	1.78 (2.33)	53.5 (12.7)	1.16 (1.83)	50.0 (10.0)	0.003	0.003	0.072	0.885
Conduct problems	1.37 (2.05)	57.0 (16.5)	0.89 (1.66)	53.0 (13.4)	0.51 (1.21)	50.0 (10.0)	<0.001	<0.001	0.156	0.123
Hyperactivity	3.50 (3.05)	54.5 (10.7)	2.97 (2.92)	52.2 (10.8)	2.35 (2.87)	50.0 (10.0)	0.002	0.001	0.258	0.277
Peer problems	1.51 (1.93)	57.1 (16.1)	1.11 (2.00)	53.8 (16.7)	0.65 (1.20)	50.0 (10.0)	<0.001	<0.001	0.123	0.302
Prosocial behavior	7.00 (2.54)	55.4 (12.3)	7.52 (2.44)	52.6 (12.0)	8.05 (2.03)	50.0 (10.0)	<0.001	<0.001	0.210	0.190

*Higher T-scores equal more difficulties and less prosocial behavior. Group differences calculated on T-scores with Welch ANOVA and post hoc Games-Howell*.

*FHR-BP, Familial high-risk bipolar group; FHR-SZ, Familial high-risk schizophrenia group*.

### Prevalence of SDQ Scores Within Clinical Range

To quantify the clinical significance of between-group differences in prevalence of SDQ scores within clinical range, we applied logistic regression analyses to compare the proportion of children from each risk group who were within the 10 percent (90th percentile) poorest scored, calculated from the control group, on each subscale and on the Total Difficulties scale. The 90th percentile from the control group was chosen as an illustrative cut-off because it approximates cut-off for significant problems in a normal population, as previously shown in other studies (13, 14, 28). Danish norms have recently been published (35) but no national cut-off score for this particular, narrow age group were presented. As our control group is a matched control group and of considerable size, we assessed it appropriate to use the studies control group to construct cut-off scores. As the individual scales are short and have a narrow score range (1–10), producing exact 90th percentile cut-offs are not feasible. We thus chose the cut-off score that encompasses the 90th percentile with the closest proximity and indicated the exact percentage of children in the control group (Table 3). We report he proportion of children scored above cut-off and the odds ratios (OR) between groups (Table 3).

**Table 3:** Proportion of children with familial high risk of schizophrenia or bipolar disorder scored within clinical range compared to that of controls.

**Primary caregiver** **ratings**	**Raw-score cut-off value closest to the 90th (10th)**^**a**^ **percentile of the PBC**		**Controls**	**FHR-SZ**			**FHR-BP**		
	**Girls**	**Boys**	**Percent in clinical range**	**Percent in clinical range**	**OR (CI)**	***p*-value**	**Percent in clinical range**	**OR (CI)**	***p*-value**
Total difficulties	≥14	≥11	9.4	28.1	3.8 (2.0–7.1)	<0.001^*^	19.4	2.3 (1.1–4.8)	0.023^*^
Emotional symptoms	≥5	≥4	7.5	24.0	3.9 (1.9–7.7)	<0.001^*^	18.4	2.8 (1.3–6.0)	0.010^*^
Conduct problems	≥2	≥2	13.8	38.3	3.9 (2.2–6.9)	<0.001^*^	23.3	1.9 (1.0–3.6)	0.051
Hyperactivity	≥7	≥6	7.5	21.6	3.4 (1.7–6.7)	<0.001^*^	14.6	2.1 (0.9-4.7)	0.073
Peer problems	≥3	≥3	6.9	20.4	3.4 (1.7–7.1)	<0.001^*^	11.7	1.8 (0.8–4.2)	0.191
Prosocial behavior^a^	≤ 6	≤ 6	10.7	16.2	1.6 (0.8–3.1)	0.151	10.7	1.0 (0.4–2.2)	0.998
**Teacher ratings**	**Raw-score cut-off value closest to the 90th (10th)**^**a**^ **percentile of the PBC**		**Controls**	**FHR-SZ**			**FHR-BP**		
	**Girls**	**Boys**	**Percent in clinical range**	**Percent in clinical range**	**OR (CI)**	***p*** **-value**	**Percent in clinical range**	**OR (CI)**	***p*** **-value**
Total difficulties	≥12	≥13	8.5	26.1	3.8 (1.9–7.7)	<0.001^*^	15.4	2.0 (0.9–4.4)	0.110
Emotional symptoms	≥5	≥4	10.6	16.4	1.7 (0.8–3.3)	0.163	17.6	1.8 (0.8–3.8)	0.133
Conduct problems	≥3	≥3	5.0	24.6	6.3 (2.7–14.7)	<0.001^*^	12.1	2.6 (1.0–7.1)	0.055
Hyperactivity	≥5	≥8	9.2	15.7	1.8 (0.9–3.8)	0.108	13.2	1.5 (0.7–3.4)	0.344
Peer problems	≥2	≥3	8.5	26.1	3.8 (1.9–7.7)	<0.001^*^	14.3	1.8 (0.8–4.1)	0.170
Prosocial behavior^a^	≤ 5	≤ 4	6.4	20.1	3.7 (1.7–8.2)	0.001	15.4	2.7 (1.1–6.4)	0.031

*SDQ clinical cut-off scores (Clinical range = T-score > 65) are calculated from the control group. Odds ratios (OR) and p-values are calculated from T-scores*.

a *The Prosocial scale is a reversed scale with higher scores equaling better performance*.

*FHR-BP, Familial high-risk bipolar disorder group; FHR-SZ, Familial high-risk schizophrenia group*.

*SDQ, The strengths and difficulties questionnaire (28)*.

* *Significant value: For the total difficulties a p < 0.05 are considered significant. For the four difficulties scales (emotional symptoms, conduct problems, hyperactivity, and peer problems) and the Prosocial Behavior scale a p < 0.01 is considered significant according to Bonferroni alfa level correction for multiple comparisons (0.05/5 = 0.01)*.

### Difference in General Functioning (CGAS) Between Children Scored Below and Above SDQ Total Difficulties Cut-off

We assessed differences in CGAS mean scores between the above and below cut-off groups by independent samples *t*-test, to qualify the assumption of an association between an SDQ Total Difficulties scale score above cut-off and poorer general functioning.

### The Discriminatory Ability of the SDQ Compared to the CBCL and TRF

We applied receiver operating characteristics (ROC) curves to determine the ability of SDQ and the CBCL total scores to distinguish between FHR children with any lifetime Axis I diagnosis vs. non-diagnosed children. ROC curve analysis was further used to determine the ability of the SDQ and CBCL hyperactivity and conduct scales to distinguish between FHR children with and without a lifetime ADHD and DBD diagnosis, respectively. Comparisons of the areas under the ROC curves for SDQ and CBCL, respectively, were performed using paired sample statistics (Table 4). All ROC analyses were performed on continuous outcome T-scores.

**Table 4:** A. Ability of SDQ-P and CBCL to discriminate between cases with and without disorders within the FHR groups analyzed by ROC curves.

**Problem scale SDQ-P/CBCL**	**Research axis I diagnosis (*N* with/without)**	**Area under curve (95% CI)**		**AUC^**c**^ SDQ-P vs. CBCL**	**Sensitivity**		**Specificity**	
		**SDQ-P**	**CBCL**	***p*-value^**a**^**	**SDQ-P**	**CBCL**	**SDQ-P**	**CBCL**
Total scale	Any axis I^b^ Total FHR cohort (101/165)	0.79 (0.73–0.84)^**^	0.78 (0.72–0.83)^**^	0.752	48.5	41.6	89.7	88.5
	FHR-SZ (63/101)	0.82 (0.75–0.89)^**^	0.80 (0.72–0.87)	0.454	57.1	46.0	90.1	88.1
	FHR-BP (38/64)	0.75 (0.65–0.84)	0.76 (0.67–0.86)	0.611	34.2	34.2	89.1	90.6
Hyperactivity scale	Any ADHD total FHR cohort (43/226)	0.92 (0.88–0.95)^**^	0.88 (0.83–0.94)^**^	0.085	69.8	67.4	90.7	88.1
Conduct scale	Any DBD total FHR cohort (16/250)	0.91 (0.85–0.97)^**^	0.88 (0.77–0.99)^**^	0.511	81.3	93.8	82.0	82.8
**Problem scale SDQ-T/TRF**	**Research axis I diagnosis (*****N*** **with/without)**	**Area under curve (95% CI)**		**AUC SDQ-T vs. TRF**	**Sensitivity**		**Specificity**	
		**SDQ-T**	**TRF**	***p*** **-value** ^**a**^	**SDQ-T**	**CBCL**	**SDQ-T**	**CBCL**
**B. Ability of SDQ-T and TRF to discriminate between cases with and without disorders within the FHR groups analyzed by ROC curves**								
Total scale	Any axis I^bc^ Total FHR cohort (86/136)	0.75 (0.68–0.82)^**^	0.76 (0.69–0.82)^**^	0.810	37.2	43.0	91.2	89.0
	FHR-SZ (55/77)	0.77 (0.69–0.85)^**^	0.78 (0.70–0.85)^**^	0.789	43.6	43.6	88.3	87.0
	FHR-BP (31/59)	0.72 (0.60–0.83)^*^	0.71 (0.59–0.83)^*^	0.764	32.3	41.9	93.2	91.5
Hyperactivity scale	Any ADHD^c^ Total FHR cohort (36/186)	0.85 (0.78–0.91)^**^	0.86 (0.80–0.91)^**^	0.685	47.2	47.2	91.4	92.5
Conduct scale	Any DBD^c^ Total FHR cohort (16/206)	0.82 (0.70–0.94)^**^	0.92 (0.87–0.97)^**^	0.021	68.8	81.3	84.5	88.8

*Only children with both an SDQ-P and an CBCL/SDQ-T and TRF, respectively were included in the analysis. Sensitivity and specificity at cut-off score closest to T-score of 65 according to the ROC curve analyses*.

* *p < 0.001*,

** *p < 0.0001. BP, Bipolar disorder; FHR, Familial high risk; CBCL, Child Behavior Checklist; SDQ, The strengths and difficulties questionnaire; -P, Parent version; -T, Teacher version; SZ, Schizophrenia; TRF, teachers report form*.

a *P-value of z-test for comparing paired area under ROC curves (AUC) of SDQ and CBCL*.

b *Any axis I excluding elimination disorders. ^c^Four children who had an SDQ-T/TRF did not have a diagnostic assessment*.

c *Area under curve*.

*ADHD, Attention deficit hyperactivity disorder; DBD, Disruptive behavior disorder*.

Results on the discriminatory abilities of the SDQ, CBCL, and TRF analyzed with ROC curves are expressed by sensitivity, specificity and area under the curve (AUC) where an AUC of 1.0 reflects perfect discrimination, and 0.5 reflects no better than chance accuracy. To aid interpretation of the results, we applied the following distinction: 0.5–0.7 = poor discrimination (coin toss), 0.7–0.8 = Acceptable discrimination, 0.8–0.9 = Excellent discrimination, >0.9 = outstanding discrimination (36). Sensitivity and specificity values closest to the T-score = 65 cut-off value is reported.

Teachers' SDQ ratings were examined by the same methods as described above. SDQ-T ROC curves were compared to the TRF, the teacher-rated equivalent to the CBCL.

### Correlation of Primary Caregiver and Teacher Rated SDQ Scores

Interrater agreement between the primary caregiver and teacher SDQ ratings and between primary caregiver and teacher rated CBCL/TRF were performed by intra-class correlation coefficient (ICC) with two-way random effect model on absolute agreement (37, 38). To guide interpretation the following classifications will be used: ICC values <0.40 are considered poor agreement, values from 0.40 to 0.59 are fair, values between 0.60 and 0.74 are good, and ICC values > 0.75 are considered excellent (39).

### Handling of Missing

According to the SDQ scoring manual, subscale scores can be scaled up pro-rata, if at least three of five items are rated (40). If a subscale had more than two missing items, the subscale for that respondent was censured. To ensure that data was not affected by this procedure, all missing values were assessed, and found to be missing at random and to represent a minute fraction of the data.

We used IBM SPSS Statistics, version 26 for ROC comparisons. All other analyses were conducted using IBM SPSS Statistics, version 25.

## Results

### Participants' Characteristics

The three groups did not differ regarding age or sex of the children. FHR children less often lived with both biological parents, and their primary caregiver had a lower level of functioning than control primary caregivers (Table 1). Distribution of sex did not differ between children participating with SDQ data and non-participants in any of the three groups. Daily life functioning (CGAS) differed between FHR-BP participants [mean (SD) = 72.1 (14.8)] and non-participants [*N* = 15 with a CGAS, mean (SD) = 83.9 (11.3)] (*p* = 0.004) but only on the SDQ-P.

### Descriptive SDQ Mean Values and Between-Group Mean SDQ T-Score Differences

The difference in mean SDQ-P T-scores between the FHR-SZ group and the control group was significant on both the Total Difficulties Scale and on all four difficulties scales (*p* < 0.001), but non-significant regarding the Prosocial Behavior scale. The FHR-BP group differed significantly from the controls on the SDQ-P Total Difficulties Scale score (*p* = 0.026), the Emotional Symptoms scale (*p* = 0.025) and the Conduct Problems scale (*p* = 0.041), but not on the remaining scales. The two FHR groups were significantly different from each other on all SDQ-P scales (*p* = 0.011–0.046) except the Emotional Symptoms scale. The FHR-BP group scored intermediate of the control group and the FHR-SZ group on all four SDQ-P difficulties scales (Table 2).

Regarding SDQ-T ratings, the FHR-SZ group differed from the control group on all scales (*p* = <0.0001–0.003), including the Prosocial Behavior scale (*p* < 0.001), whereas the FHR-BP group only differed from the control group on the Total Difficulties Scale (*p* = 0.048). However, the two FHR groups did not differ from each other on any scales of the SDQ-T (Table 2).

### Prevalence of SDQ Scores Within the Clinical Range

FHR-SZ children were more likely to have an SDQ-P Total difficulties Scale score within clinical range compared to control children (28.1 vs. 9.4%) (OR = 3.8, CI 2.0–7.1, *p* ≤ 0.001). This was also true for FHR-BP children (19.4 vs. 9.4%) (OR = 2.3, CI 1.1–4.8, *p* ≤ 0.023). On all SDQ-P difficulties scales, FHR-SZ children had higher odds of significant difficulties compared to the control children (*p* ≤ 0.001). Odds of FHR-BP children only differed from that of the control children on the SDQ-P Emotional Symptoms scale (*p* = 0.010). Neither of the FHR groups differed from the control group regarding the SDQ-P Prosocial Behavior scores (Table 3).

SDQ-T identified a lower proportion of FHR children within clinical range than the SDQ-P except regarding peer problems and less prosocial behavior. Further, the OR of conduct problems for FHR-SZ was high (OR = 6.3, CI 2.7–14.7, *p* ≤ 0.001) compared to controls according to the SDQ-T.

### Difference in General Functioning (CGAS) Between Children Scored Below and Above SDQ Total Difficulties Cut-off

CGAS mean scores were higher for the “below SDQ-P cut-off groups” [*N* = 347, mean (SD) = 75.8 (13.2)] compared to the “above SDQ-P cut-off group” [*N* = 82, mean (SD) = 55.9 (12.1)] with a statistically significant difference of 19.9 points, 95% CI [16.8, 23.0], *t*_(427)_ = 12.446, *p* = 0.0001. Results for the individual high-risk groups as well as all SDQ-T results were very similar to these results (Supplementary Table 1).

### The Discriminatory Ability of the SDQ Compared to the CBCL and TRF

The ability of the SDQ-P and the CBCL total scores to distinguish between children with and without any lifetime Axis I diagnosis was acceptable to excellent and non-significantly different (Table 4). The ability of the SDQ-P hyperactivity scale to discriminate between children with and without a diagnosis of Attention-Deficit Hyperactivity Disorder (AUC = 0.92, CI 0.89–0.95) and the conduct scale in regards to Disruptive Behavior Disorder (AUC = 0.93, CI 0.89–0.98), were both outstanding and non-significantly different from that of the corresponding CBCL scales (Table 4A). Sensitivity of the SDQ-P and CBCL ranged from 34.2% for any Axis I in the FHR-SZ group to 81.3 and 93.8%, respectively, for any disruptive behavior disorder, the latter showing the highest discrepancy between SDQ-P and CBCL. Specificity ranged from 82 to 90.6%.

The discriminatory abilities of SDQ-T and TRF resemble the results of the SDQ-P and CBCL (Table 4B).

### Correlations of Primary Caregiver and Teacher Rated SDQ Scores

Interrater agreement between primary caregiver and teacher on the Total Difficulties Scale was good and regarding the individual SDQ difficulties scales ICC values were primarily in the fair to good range. CBCL and TRF interrater agreement were likewise in the fair to good range (Supplementary Table 2).

## Discussion

In this large representative, register constructed, population based cohort of 7-years-old children at familial high-risk of schizophrenia or bipolar disorder and controls, we observed that a substantially larger proportion of children with a familial high risk of schizophrenia and of those with a familial high risk of bipolar disorder had significant daily life behavioral difficulties compared to matched population based control children as evaluated both by their primary caregivers and their schoolteachers on the Strengths and Difficulties Questionnaire. Further, children who were found above cut-off for clinically significant difficulties had a markedly lower level of daily life functioning measured with CGAS compared to children below cut-off. Teachers identified less prosocial behavior in both FHR groups. The discriminatory abilities of the SDQ were found to be good, with high AUC values and moderate to high sensitivity and high specificity values. Moreover, the SDQ was compellingly equating to the well-recognized, but more time-consuming CBCL and TRF. Interrater agreement of the caregiver and the teacher-rated SDQ fell in line with results of other studies which in general have found reliable agreement on all difficulties scales and best agreement on the total scale and the hyperactivity scale (13, 15, 28).

Identifying that between one fifth of 7-year-old FHR-BP children and one third of FHR-SZ children scored above threshold for emotional and behavior problems indicative of mental illness is a prominent finding that deserves attention.

Mean scores and the cut-off scores, based on our cohorts' control group, are somewhat lower than reported from other European countries (41). Nevertheless, for girls, they are in line with previous investigations of the general Danish population (35, 41) and other Scandinavian countries (41) as well as in line with cut-off scores determined from ROC analysis and 90th percentiles in a Swedish sample (42), thus supporting the validity of our findings. However, our cut-off scores for boys tend to be lower than previous findings both in Danish and in other Scandinavian settings with caregiver ratings having the most considerable discrepancy. Nonetheless, we find that an equal proportion of boys and girls are scored above cut-off in the FHR-BP group both according to caregiver ratings and teacher ratings. Only a significantly larger proportion of FHR-SZ boys are above cut-off, and this is only according to caregiver ratings. If boys from the control group should somehow be “better” functioning than average, we would have expected that a substantially larger proportion of boys than girls in both the two FHR groups would be scored above cut-off for significant difficulties by both teachers and caregivers. We thus believe that the finding of a relatively high proportion of children in the two high-risk groups with significant difficulties suggestive of mental illness is a true finding that warrants pre-emptive measures early in life for these at-risk children. Further strengthening the validity of our results is that the above cut-off group has a markedly lower level of functioning than the below cut-off group.

Our secondary aim was to investigate whether a short and accessible measure of children's behavioral difficulties could distinguish between children with and without a psychiatric diagnosis determined by a thorough diagnostic interview, the K-SADS. The results from ROC analysis indicate that the SDQ has a compelling ability to discriminate between children with and without a psychiatric diagnosis with moderate to high sensitivity and high specificity. These results and the noticeable equality between discriminatory abilities of the SDQ and the CBCL resemble previous findings (19, 42–45) supporting the reliability of the present findings.

Collectively, these results indicate that the SDQ functions well as a screening tool for the identification of children at FHR for SZ and BP in need of aid. These results are from a time point in the FHR children's lives were they have not yet developed any severe mental illness, but some do already have other psychiatric disorders and many have lower levels of functioning than controls (3) as well as affected cognition (46). It is, however, essential to remember that this is a cross sectional study and that the results are a snapshot of status at age seven. We do not yet know the future development of these children or if the current identified problems are transitory although other studies indicate a predictive value of SDQ scored at age 5–7 and the presence of mental illness at age 11–12 (19, 20).

Our focus was on the primary caregiver rated SDQ, but analyses of the teacher rated SDQ revealed very similar results. This, together with moderate correlations between primary caregiver and teacher SDQ ratings, and also notably higher than what has been found in other studies (13, 47), strengthens the robustness of the study results. Teachers generally rated less difficulties than primary caregivers except for on the Hyperactivity scale and the Peer Problem scale, where teachers rated both high-risk groups as having more problems. When comparing results of SDQ teacher and primary caregiver ratings, it is important to note that the teacher and primary caregiver rated SDQ cohorts are not entirely overlapping. Further, it has to be acknowledged that primary caregiver ratings could be affected by the presence of mental illness in the caregiver, him- or herself, as this is a factor known to potentially distort reporting's (47, 48). However, the results of the present study are in accordance with other studies where primary caregivers also rated problems more severely than teachers and teachers also were more prone to rate externalizing problems like hyperactivity and problems regarding social interaction with peers (49).

## Strengths and Limitations

A significant strength of this study is that we applied a multi-informant method in a register-based sample of 522 same age children. Assessing children in different social settings gives valuable information of behaviors potentially related to mental illness. We explored emotional and behavioral problems both by multi-informant and by a multimethodological (questionnaires, interviews, lab-tests, etc.) approach, thus giving us a unique opportunity to compare instruments. Importantly SDQ and CBCL were not calculated previous to or used explicitly in connection to evaluation of child diagnosis, but single items of both the SDQ, CBCL and TRF could potentially in some cases have been consulted in relations to the diagnostic process thus rendering the diagnosis not completely independent of the questionnaires.

Further, we estimated cut-off points indicative of significant difficulties constructed from our control group, which had the qualities of not only being matched to the FHR-SZ children but also being population based. Further strengthening the present study is that all children were within a narrow age range and drawn and matched through registers from a national cohort of eligible children. We thus did not have a problem of referral bias and the children were included regardless of their parent's current stage of illness and potential contact to the psychiatric system. Also strengthening the study is the high participation and completion rate of families in both the VIA 7 Study as a whole and in the present study. The group of participating FHR-BP families in the VIA 7 Study was from the outset planned to be smaller than the two other groups partly due to economic and practical issues. With more participants in the FHR-BP group, we might have found significant results on more scales for example the conduct problem and hyperactivity scales. More participants would undoubtedly have strengthened the study and we have to consider the risk of having committed a Type II error. Replication of the study in a larger scale could thus be of value. Because of the low number of siblings and double high-risk children (i.e., children with two parents with severe mental illness), we did not include the effects of siblings or double high-risk children in the analyses. The influence of genetic similarity and genetic load, respectively, was thus not included in the statistical model and we cannot rule out a possible effect.

The results regarding the discriminatory and predictive abilities of the SDQ and comparisons to the abilities of the CBCL and TRF, are necessarily preliminary as the study is limited to a very narrow age group. However, previous studies with other study groups and age ranges have arrived at similar conclusions (19, 21, 22, 42, 43, 45, 50).

## Conclusion

We aimed to assess whether children at familial high risk of schizophrenia or bipolar disorder had more daily life behavioral difficulties indicative of mental illness compared to control children. We observed that the SDQ not only identified a significant proportion of familial high-risk children with substantial everyday life difficulties indicative of mental illness, but also that these children had markedly affected daily life functioning. Further, the SDQ was able to discriminate between FHR children with and without a diagnosis with compelling accuracy and undistinguishable from the lengthier CBCL. The SDQ is a short and accessible measure assessing both strengths and difficulties of children, rendering it highly acceptable for both parents and teachers. The discriminatory abilities of the SDQ found in the present study, along with its general acceptability to respondents, it's brevity as well as low costs of administration and evaluation, renders the SDQ a compelling screening instrument for the identification of children with potential mental illness. This, both in research, clinical and importantly, in pre-clinical settings were the SDQ could serve as an epidemiological screening tool of large groups of FHR children.

Collaboration between child and adolescent psychiatry and adult psychiatry as well as schools, could allow early identification and interventions targeting FHR children who are exhibiting problematic behavior indicative of mental illness. Early identification could give these at-risk children a chance to turn their development in a more positive direction and as a result of this alleviate adverse consequences.

## Data Availability Statement

The datasets analyzed for this study can be accessed upon request to the corresponding author.

## Ethics Statement

The Danish High Risk and Resilience Study - VIA 7 was approved by the Danish Data Protection Agency (J.nr.: 2012-58-0004). The Danish National Committee on Health Research Ethics evaluated the study and concluded that further approval was not required due to the observational nature of the study.

## Author Contributions

KS wrote the manuscript and performed data analysis with input from AT, DE, NH, CC, BB, DG, AG, MG, OM, MN, JJ, CO, and KP. KS, DE, NH, CC, BB, AG, DG, and MG collected the data. All authors contributed to the study conception, design, critically revised the manuscript, and approved the final version.

## Funding

This work was supported by TrygFonden, the Mental Health Services of the Capital Region of Denmark, the Lundbeck Foundation Initiative for Integrative Psychiatric Research (iPSYCH), Aarhus University, and the Beatrice Surovell Haskell Fund for Child Mental Health Research of Copenhagen.

## Conflict of Interest

The authors declare that the research was conducted in the absence of any commercial or financial relationships that could be construed as a potential conflict of interest.

## Publisher's Note

All claims expressed in this article are solely those of the authors and do not necessarily represent those of their affiliated organizations, or those of the publisher, the editors and the reviewers. Any product that may be evaluated in this article, or claim that may be made by its manufacturer, is not guaranteed or endorsed by the publisher.

## Abbreviations

ADHD: Attention-Deficit/Hyperactivity Disorder
CBCL: The Child Behavior Checklist school-age version
CGAS: The Children's Global Assessment Scale
DBD: Disruptive Behavior Disorder
FHR: Familial High Risk
FHR-BP: Familial high risk for Bipolar disorder
FHR-SZ: Familial High Risk for Schizophrenia
ICC: Intra-class Correlation Coefficient
K-SADS-PL: The Schedule for Affective Disorders and Schizophrenia for School-Age Children—Present and Lifetime Version
ROC: Receiver Operating Characteristics
SDQ: The Strengths and Difficulties Questionnaire
The VIA 7 Study: The Danish High Risk and Resilience Study—VIA 7
TRF: The Teacher's Report Form.
