# Supplementary material for: The Strengths and Difficulties Questionnaire Is of Clinical Significance Regarding Emotional and Behavioral Problems in 7-Year-Old Children With Familial Risk of Schizophrenia or Bipolar Disorder and Population-Based Controls the Danish High Risk and Resilience Study–VIA 7; A Population-Based Cohort Study
Source: Front Psychiatry. 2022 May 25;13:861219. doi: 10.3389/fpsyt.2022.861219 (PMC9174569; doi:10.3389/fpsyt.2022.861219)
Supplement: Supplementary file 1 [file Table_1.DOCX]

| Table S1. CGAS mean scores of the SDQ Total Difficulties below and above cut-off groups. | | | | | | | | | |
| --- | --- | --- | --- | --- | --- | --- | --- | --- | --- |
| **PRIMARY CAREGIVER RATINGS** |  | | | | |  | | | *p*-value |
|  | Below Cut-off group | |  | Above Cut-off group | |  |  |  |  |
|  | N | CGAS mean (SD) |  | N | CGAS mean (SD) |  | Mean difference (95% CI) |  |  |
| Total cohort | 347 | 75.8 (13.2) |  | 82 | 55.9 (12.1) |  | 19.9 (16.8-23.0) |  | <.0001 |
| FHR-SZ | 120 | 72.7 (13.5) |  | 47 | 53.7 (11.7) |  | 19.1 (14.7-23.5) |  | <.0001 |
| FHR-BP | 83 | 75.5 (13.2) |  | 20 | 57.6 (12.1) |  | 18.0 (11.6-24.4) |  | <.0001 |
| Controls | 144 | 78.6 (12.5) |  | 15 | 60.9 (12.4) |  | 17.7 (11.0-24.3) |  | <.0001 |
| **TEACHER RATINGS** |  | | | | | | | | *p*-value |
|  | Below Cut-off group | |  | Above Cut-off group | |  |  |  |  |
|  | N | CGAS mean (SD) |  | N | CGAS mean (SD) |  | Mean difference (95% CI) |  |  |
| Total cohort | 303 | 75.3 (13.8) |  | 59 | 55.6 (12.6) |  | 19.7 (15.9-23.5) |  | <.0001 |
| FHR-SZ | 99 | 70.5 (15.6) |  | 34 | 55.2 (12.1) |  | 15.3 (9.5-21.1) |  | <.0001 |
| FHR-BP | 76 | 75.8 (12.5) |  | 14 | 52.6 (12.7) |  | 23.2 (15.9-3054) |  | <.0001 |
| Controls | 128 | 78.6 (12.0) |  | 11 | 60.6 (13.5) |  | 18.0 (10.5-25.6) |  | <.0001 |
| SDQ: The Strengths and Difficulties Questionnaire. CGAS: Children’s Global Assessment Scale.  FHR-BP: Familial high-risk bipolar group FHR-SZ: Familial high-risk schizophrenia group | | | | | | | | | |
