# Supplementary material for: The Strengths and Difficulties Questionnaire Is of Clinical Significance Regarding Emotional and Behavioral Problems in 7-Year-Old Children With Familial Risk of Schizophrenia or Bipolar Disorder and Population-Based Controls the Danish High Risk and Resilience Study–VIA 7; A Population-Based Cohort Study
Source: Front Psychiatry. 2022 May 25;13:861219. doi: 10.3389/fpsyt.2022.861219 (PMC9174569; doi:10.3389/fpsyt.2022.861219)
Supplement: Supplementary file 2 [file Table_2.DOCX]

| Table S2. Inter-rater agreement between. | | | | | | | | | | | | |
| --- | --- | --- | --- | --- | --- | --- | --- | --- | --- | --- | --- | --- |
| SDQ-P/SDQ-T | ICC (95%CI) | | | | | | | | | | | |
|  |  | Total cohort |  |  | FHR-SZ |  |  | FHR-BP |  |  | Controls |  |
| N | 347 | | | 129 | | | 85 | | | 133 | | |
| Total Difficulties scale | .68 (.60-.74)** | | | .67 (.54-77)** | | | .64 (.44-.77)** | | | .60 (.44-.72)** | | |
| Emotion scale | .46 (.33-.56)** | | | .36 (.10-.54)* | | | .57 (.34-.72)** | | | .34 (.08-.53)* | | |
| Conduct scale | .62 (.53-.69)** | | | .64 (.49-.75)** | | | .52 (.26-.69)** | | | .50 (.39-.65)** | | |
| Hyper scale | .71 (.64-.77)** | | | .77 (.68-.84)** | | | .58 (.36-.73)** | | | .69 (.53-.77)** | | |
| Peer scale | .56 (.45-.64)** | | | .33 (.05-.53)* | | | .72 (.57-.82)** | | | .64 (.49-.74)** | | |
| Prosocial scale | .31 (.14-.45)** | | | .40 (.14-.59)** | | | .19 (-.17-.45)^NS^ | | | .17 (-.14-.40)^NS^ | | |
| CBCL/TRF | ICC (95%CI) | | | | | | | | | | | |
|  |  | Total cohort |  |  | FHR-SZ |  |  | FHR-BP |  |  | Controls |  |
| N | 479 | | | 160 | | | 96 | | | 159 | | |
| Total scale | .63 (.55-.69)** | | | .64 (.51-.74)** | | | .53 (.30-.69)** | | | .58 (.43-.69)** | | |
|  |  | | |  | | |  | | |  | | |
| Conduct scale | .68 (.62-.74)** | | | .67 (.55-.76)** | | | .70 (.55-.80)** | | | .53 (.36-.66)** | | |
| Hyper scale | .60 (.52-.68)** | | | .64 (.51-.73)** | | | .53 (.30-.69)** | | | .58 (.42-.69)** | | |
| ICC two-way random effects model absolute agreement type **p-*value <.05. ***p*-value <.001 ns = not significant. SDQ: The Strengths and Difficulties Questionnaire (R. Goodman, 2001) | | | | | | | | | | | | |
